# Comparison of fully coated anti-reflux metal stenting and per-oral endoscopic myotomy in patients with achalasia: a propensity score-matched retrospective study

**DOI:** 10.1186/s12876-022-02282-1

**Published:** 2022-05-18

**Authors:** Yu-fen Tang, Peng Jin, Yu-rong Tao, Hui Xie, Xin Wang, Dongliang Yu, Shan Tang, Jian-qiu Sheng

**Affiliations:** 1 Department of Gastroenterology, The Seventh Medical Center of PLA General Hospital, Nanmencang 5#, Dongcheng District, Beijing, 100700 China; 2 Senior Department of Gastroenterology, The First Medical Center of PLA General Hospital, Beijing, China

**Keywords:** Fully coated anti-reflux metal stenting, Per-oral endoscopic myotomy, Achalasia

## Abstract

**Background:**

Achalasia is a rare primary esophageal motility disorder disease. It is reported that the long-term effect of fully coated anti-reflux metal stent (FCARMS) implantation is satisfactory. Operated by a skilled and experienced endoscopist, the effect of per-oral endoscopic myotomy (POEM) treatment is equivalent to that of surgical myotomy. So far, there is still few evidence to prove FCARMS implantation or POEM which is better for achalasia. The choice of treatment for achalasia is still controversial. The aim of this study is to find a more suitable therapy for achalasia by comparing the efficacy of FCARMS implantation and POEM.

**Methods:**

A propensity score (PS) matching (1:2) was used in this retrospective cohort study. Data collected from consecutive patients of Achalasia, receiving FCARMS implantation or POEM therapy at the department of gastroenterology, the Seventh Medical Center of the Chinese People’s Liberation Army General Hospital from May 2007 to May 2018. According to their previous treatment, they are divided into two groups, FCARMS group and POEM group. Clinical efficacy and complications were compared between the two groups.

**Results:**

A total of 166 cases were collected, including 113 cases of FCARMS and 53 cases of POEM. By PS matching, 150 patients were enrolled (100 cases of FCARMS and 50 cases of POEM). By comparison, the FCARMS group has shorter operation time, shorter fasting time and lower hospitalization costs than the POEM group (*p* < 0.05). Common complications in the FCARMS group are nausea, vomiting, and stent shift. Repetitions of gastroscopy in the FCARMS group was more often, which were 3.8 ± 2.4 (vs 2.1 ± 1.8 of POEM) (*p* = 0.00 < 0.05) The 6-month remission rates of the FCARMS combination POEM group were 89% and 94%, respectively (*p* = 0.39), and the 2-year remission rates were 61% and 90%, respectively (*p* = 0.00).

**Conclusions:**

Stent placement is a cost-effective and safe treatment option for achalasia. The short-term effect (less than 6 months) of FCARMS is similar to that of POEM, the long-term effect (more than 2 years), POEM is better than FCARMS. HRMIIis most suitable for POEM treatment. It indicate that Patients can choose treatment methods according to their own conditions.

## Background

Achalasia is a rare primary esophageal motility disorder. Due to the lack of ganglion cells in the lower esophageal sphincter, the peristalsis of the lower esophagus disappears and the lower esophageal sphincter (LES) cannot relax [1]. It is characterized of dysphagia, posterior sternal pain, regurgitation and weight losing, etc. However, there is currently no cure for achalasia [2, 3]. The current treatment methods are mainly to reduce the pressure of the lower esophageal sphincter and relieve symptoms. Medications have little effect. The two commonly used drugs are nitrate and calcium channel blockers [4]. Laparoscopic Heller myotomy (LHM) is a surgical operation, with a success rate about 90% but adverse outcomes of Gastroesophageal reflux disease (GERD) achieved 25% and had to resort to acid-reducing medications [5], beyond complications of trauma, scars, esophagus or gastric perforation [6]. Endoscopy treatments, such as Type A botulinum toxin injection, pneumatic dilation (PD), stents implantation and POEM have been used in recently. PD needs to be repeated every 2–4 weeks, about only one in third patients can reach 5 years remittance [7–10]. Type A botulinum toxin is a biological neurotoxin released by Clostridium botulinum, which blocks the release of acetylcholine from voluntary and involuntary muscle nerve endings. The cost is higher than PD, the initial effect is better, the repeated effect is poor, and the maintenance time is short, about 6–9 months [11, 12]. It’s reported that stenting implantation treatment can relieve nearly 100 percent of achalasia patients for more than 8 years. Because the stent expands with uniform force, it is considered a safe and effective treatment [13].

POEM is a new endoscopy technique for the treatment of achalasia originating from 2008. POEM can be used also in esofageal conditions other than achalasia [14]. It is a form of per natural orifice transluminal endoscopic surgery that is completed by creating a submucosal tunnel in the lower part of esophagus to reach the inner circular muscle bundles of the LES to perform myotomy, while preserving the outer longitudinal muscle bundles. The short-term effect has been confirmed. However, it is still unclear which is better for achalasia, stenting or POEM [15]? In this study, we will compare these two methods and find answers.

## Methods

### Patients

Data collected from consecutive patients of Achalasia, definitively diagnosed by barium meals, endoscopy, and/or esophageal high resolution manometry, not treat with other methods, treated only with FCARMS implantation or POEM (treatment selected according to patients’ wishes) in the department of gastroenterology of the Seventh Medical Center of the Chinese People’s Liberation Army General Hospital from May 2007 to May 2018. According to their previous treatment, they are divided into two groups, FCARMS group and POEM group. Divided into different subtypes according to gastroscopy, High-resolution manometry (HRM), and barium esophagram. This study protocol was approved by the Ethics Research Committee of the 7th medical center of the People’s Liberation Army General Hospital, and the written informed consent of the person or his parents or legal guardians was obtained before each study.

### Equipment and procedure

#### Stent insertion and removal procedure

Stents insertion and removal were performed under monitored anesthesia (propofol). First, gastroscopy estimates the distance from the esophagus-gastric junction to the incisor, then patients received FCARMS (MTN-SE C-membrane; 80-90 mm; 18-20 mm; MicroTech, Beijing, China) implantation, with anti-reflux silicone valve in distal and recycling wire in proximal. Patients had semisolid food on the day after stent placement and were given proton-pump inhibitor to prevent reflux esophagitis. Retrieval of the stent was performed with the help of a gastroscopy 3–7 days after stent placement. The stent was grasped by the retrieval lasso or the proximal stent wire and gently pulled out.

#### POEM

All POEM procedures were performed by Dr. Peng Jin and Dr. Jianqiu Sheng. The technique is based on previously described by Inoue et al. [16]. With endotracheal intubation, general anesthesia and CO2 insufflation. First, a sub-mucosal injection with normal saline, sodium hyaluronate and indigo carmine were made 5–10 cm above the gastroesophageal junction (GEJ), followed by a 2 cm longitudinal incision. Second, the submucosal layer was dissected to make a tunnel along the esophagus and across the GEJ 2–3 cm into the proximal stomach. Third, myotomy was started 3 cm below the tunnel entrance and extended 2–3 cm into the proximal stomach. The circular muscle fiber was dissected and the longitudinal muscle fiber was preserved. Finally, the submucosal entry was closed by metal clips. The esophageal myotomy length was measured above the LES. The gastric length was measured below the GEJ. After the procedure, patients received antibiotics and intravenous nutrition for 3 days, after which they began to take liquid food and gradually changed to solid food.

#### Clinical symptom alleviation evaluation

Clinical symptoms classification and efficacy are based on Eckardt Score of the questionnaire. The questionnaire will be collected by telephone or WeChat interview or outpatient service at 6 months after treatment, and then follow-up every year. The Eckardt score was used to assess the severity of achalasia symptoms as described by Eckardt [17]. It is based on four main symptoms, dysphagia, regurgitation, chest pain and weight loss. The final score is the sum of the four scores. Clinical curative effect was judged as follows [18, 19]: Eckardt score not more than 3 points was divided into remission, while not less than 4 points was divided into treatment failure; Patients in remission of more than 6 months after FRARMS, who received retreatments, were considered treatment failures. If patients received other treatment or re-implant the stent after shorter than half a year of relief, even if Eckardt score is less than 4, it will be classified as treatment failure either. It was considered successful if stents migrated in the stomach when final Eckardt socre less than 3 points.

### Classification

#### High resolution esophageal manometric (HRM) classification

**Fig. 1:**
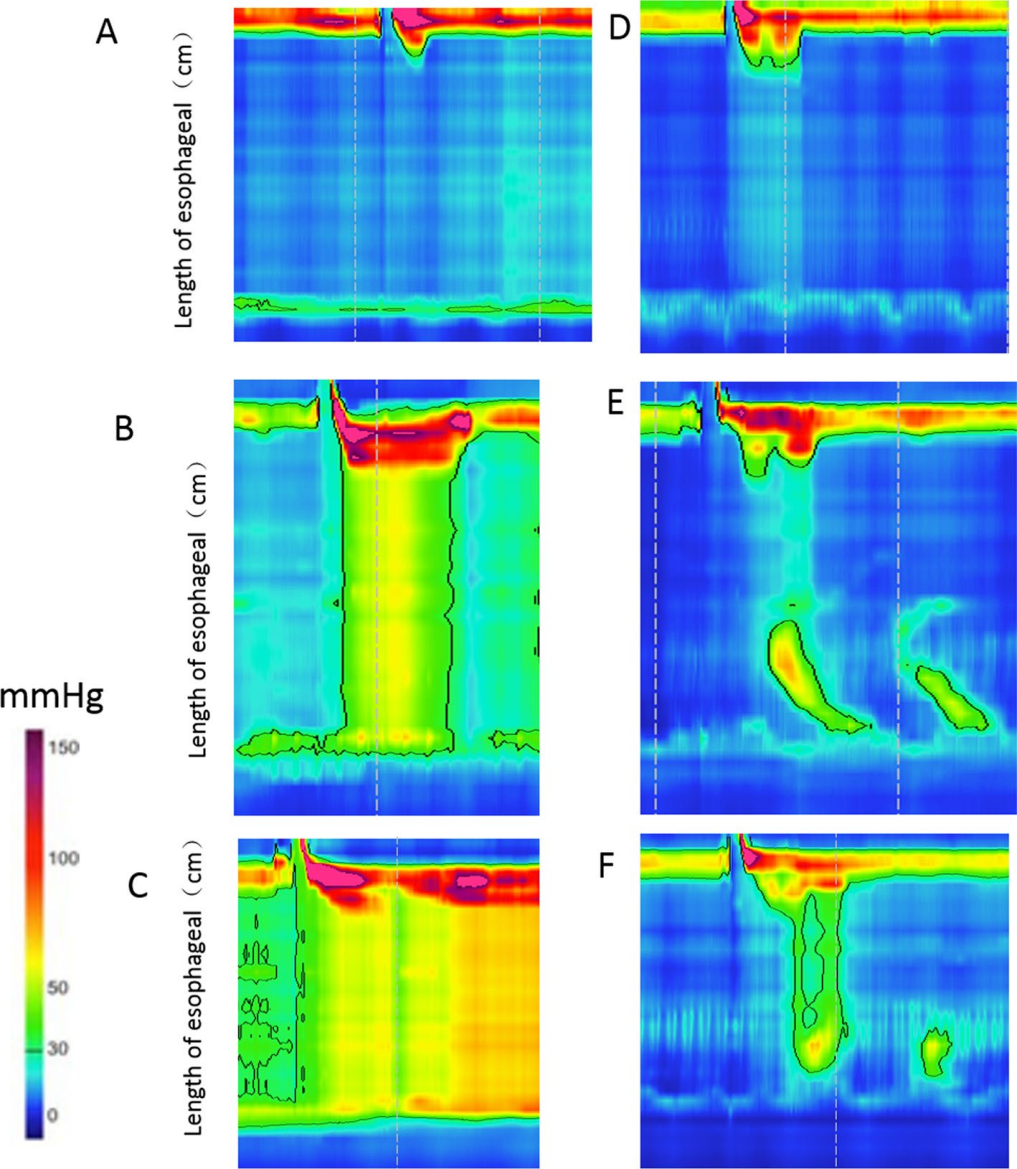
Typical pictures of three types of HRM classification before and after stent treatment. **A** Type I achalasia before treatment, no esophageal contraction and no esophageal pressurization. **B** Type II achalasia before treatment, is characterized by panesophageal pressurization and absence of a peristaltic contraction. **C** Type III achalasia before treatment, there are at least 20% premature contractions, defined as DL < 4.5 s. **D** Type I achalasia after treatment, low esophageal pressure decrease after stent insertion for 1 month and removed. **E** Type II achalasia after treatment, panesophageal pressure reduced, peristaltic contraction appeared. **F** Type III achalasia after stent treatment, Spastic hypertension disappeared

In 2008, the Chicago Classification was developed and researchers using HRM subdivided achalasia into three types. Type I, classic, is defined as achalasia with minimal esophageal pressurization, type II is defined as achalasia with panesophageal pressurization, and type III is defined as achalasia with spasm and premature contractions [20]. Patients received HRM (Sierra Scientific Instruments, San Diego, CA, USA) would be divided into three subtypes, which was according to modified Chicago Classification v3.0 [21] (Fig. 1).

#### Ling classification

**Fig. 2:**
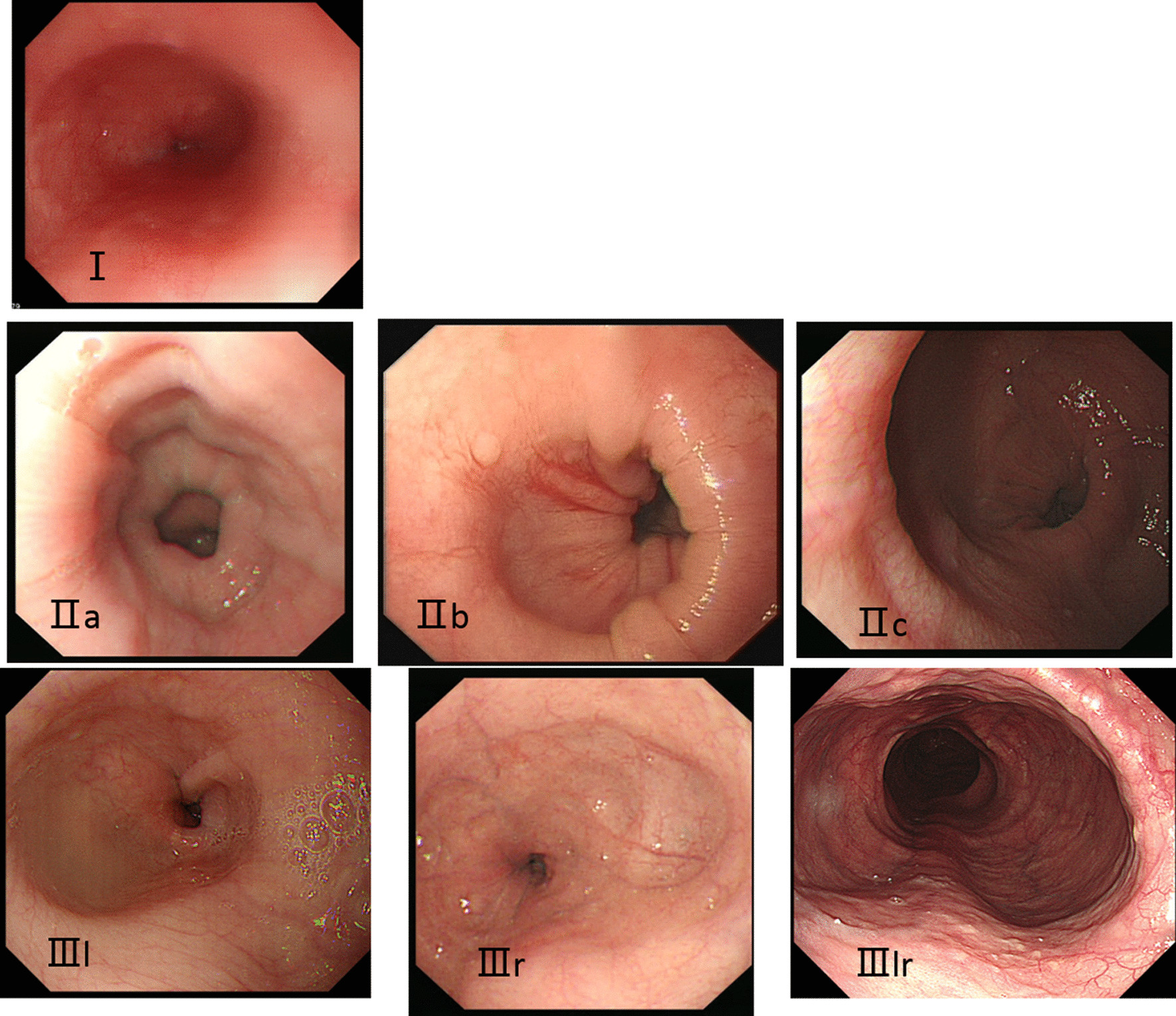
Typical pictures of each type or subtype in Ling classification. **A** Type I; **B** Type IIa; **C** Type IIb; **D** Type IIc; **E** Type IIIl; **F** Type IIIr; **G** Type IIIlr

Patients divided into three subtypes of Ling classification as described by Linghu EQ etc. [22]. Type I, smooth without multi-ring, crescent-like structure or diverticulum structure; type II, with multi-ring or crescent-like structure but without diverticulum structure; and type III, with diverticulum structure (Fig. 2).

#### Esophagography classification

**Fig. 3:**
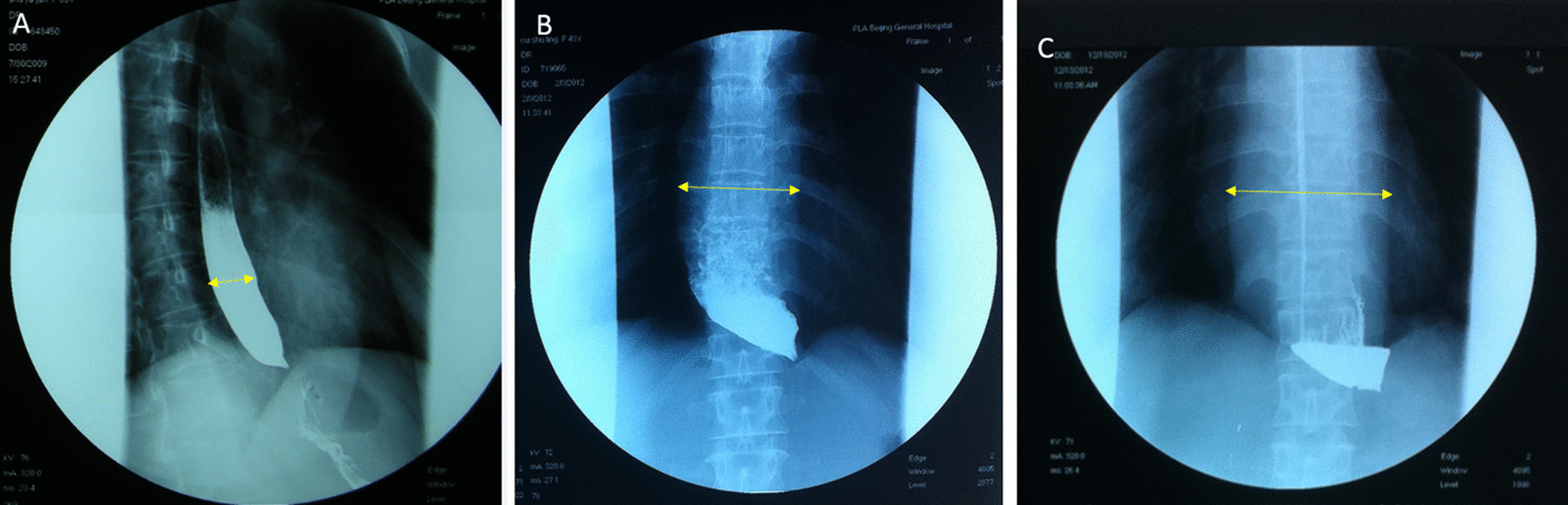
Typical pictures of Barium esophagram gradings. **A** Grade I; **B** Grade II, **C** Grade III

Barium swallow can help find aperistalsis, dilation of the esophagus, bird-beak appearance of the gastro-esophageal junction and delayed contrast medium emptying [23, 24]. Patients, according to the esophageal lumen maximal diameter, were divided into three levels: grade I, diameter < 3.5 cm, expansion involving only lower esophagus; grade II, diameter between 3.5 and 6.0 cm, expansion involving one third of lower esophagus; grade III diameter > 6.0 cm, expansion involving two third of lower esophagus(Fig. 3).

### PS matching

The propensity score (PS) is the probability that a patient will receive the treatment being tested. In a 1:1 randomized trial, this is exactly 0.5. In a nonrandomized study, this probability for each patient was unknown and depended on patient characteristics. Therefore, PS must first be estimated from existing data. A logistic regression model was used with the assigned treatment as the dependent variable and the patient characteristics before treatment as the dependent variable [25].

In PS matching, each patient treated with POEM is allocated two patients treated with FCARMS (in 1:2 matching), with the same PS that differs only slightly, within previously defined limits. The treatment effect is then estimated in the matched population, while accounting for the matching process in the statistical analysis.

### Statistical analysis

All statistical analyses were performed using SPSS version 26.0 (Chicago, USA). Use PS matching scoring method to reduce interfering factors such as age, gender, course of disease, LING type, barium esophagram type, Eckardt score and dysphagia score before treatment. Propensity sore match [25] (1:2) with caliper of 0.1 were used to delete non-matched data case in order to reduce confounding factors. Data conform to the normal distribution are expressed as Mean ± SD. The independent t test was used to compare the operating time, fasting time, postoperative hospital stay, average hospitalized cost, dysphagia score and weight-gaining between the two groups. Pearson chi-square test was used to compare percentages of curative efficiency and complications among the groups. In all instances, *p* value < 0.05 is considered significant differences.

## Results

**Fig. 4:**
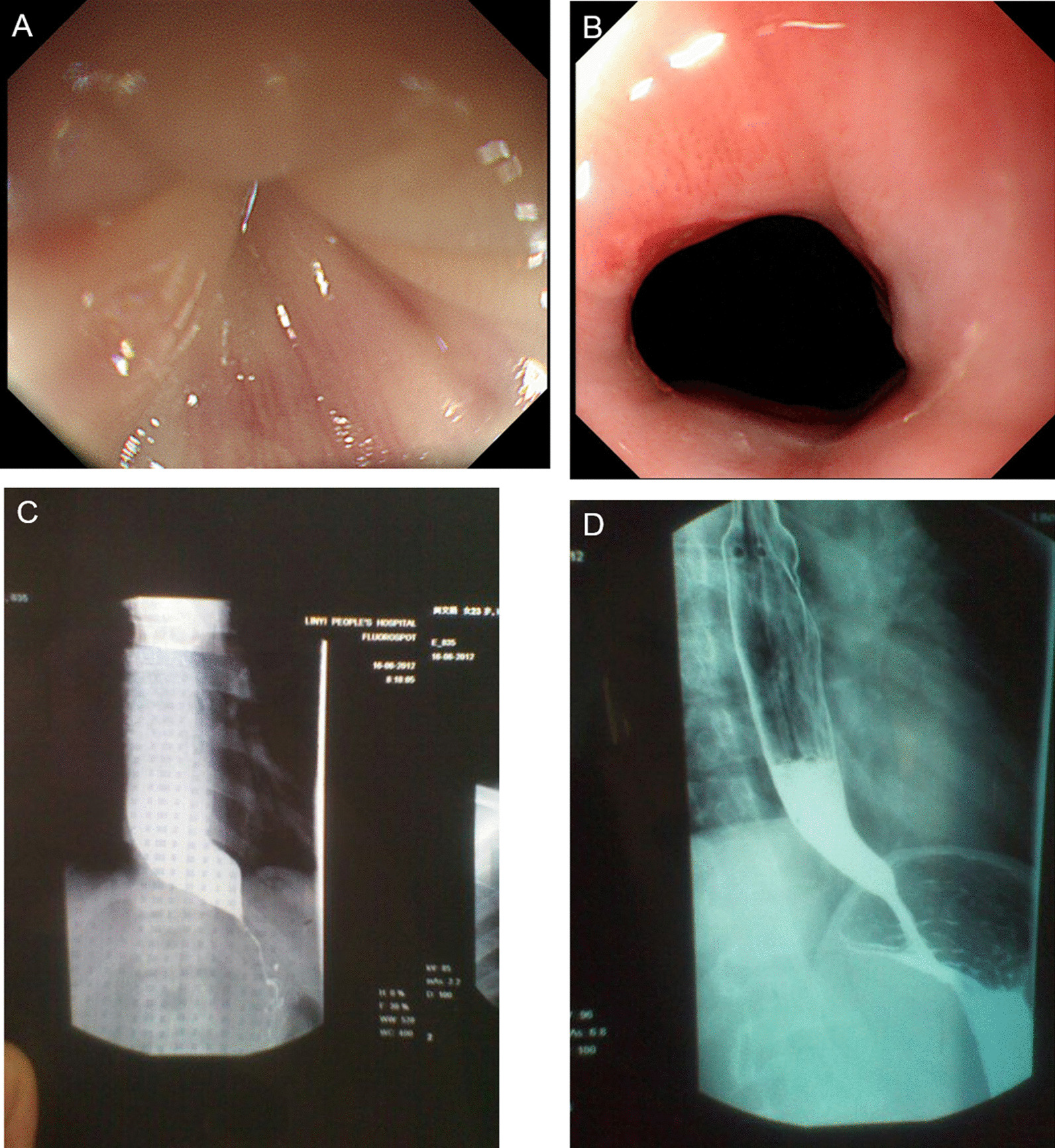
Typical pictures before and after receiving stenting treatment. **A** Cardia satus before treatment under endoscopy. **B** Cardia satus after treatment under endoscopy. **C** Beak shape under barium esophagram before treatment. **D** Barium smoothly through the cardia after treatment

A total of 166 cases, after PS matching, left 150 (FCARMS group n = 100, POEM group n = 50) patients into the study. There was no difference between the two groups in general age, gender, course of disease, Eckardt score and dysphagia score before treatment (*p* = 0.65, 1.00, 0.44, 0.40, 1.00 > 0.05) (Table 1). There are three subtypes of HRM, LING, and Barium esophagography classifications. There is no significant difference in the number of patients in each subtype between the two groups. (*p* = 0.31, 0.60, 0.27, > 0.05) (Table 1). The results of HRM and Ling’ subtype classifications didn’t affect remission rate (*p* = 0.20, 0.58, 0.94; *p* = 0.37, 0.67, 0.63 > 0.05) (Table 4). By contrast, the FCARMS group requires shorter operating time, shorter fasting time and lower hospitalization costs than the POEM group (8.41 ± 4.88 VS 73.94 ± 41.61 min, 2.24 ± 1.65 vs 4.58 ± 2.50 day, ¥ 17,787.85 ± 3711.69 vs 27,705.41 ± 8868.09, *p* = 0.00, 0.00, 0.00 < 0.05) (Table 2). The repetition times of gastroscopy in the two groups were 3.8 ± 2.4 and 2.1 ± 1.8 times respectively, the difference was significant (*p* = 0.00 < 0.05). The more complications in the FCARMS group were nausea, vomiting, and stent displacement (*p* = 0.00, 0.00 < 0.05). While in the POEM group, it had a higher perforation incidence (*p* = 0.01 < 0.05). The other complications of bleeding (In stent group was manifested as delayed hemorrhage, hematemesis or vomiting of coffee-like substance after treatment. In severe cases, the amount of bleeding was about 200–300 ml), fever, chest pain and abdominal pain had no significant difference between the two groups (*p* = 0.34, 0.34, 0.36, 0.13 > 0.05) (Table 2). At 6 months, the dysphagia was significantly relived in both groups (*p* = 0.01 < 0.05) (Table 2) (Fig. 4). The 6-month remission rates of the FCARMS combination POEM group were 89% and 94%, respectively (*p* = 0.3. At 1 and 2 years follow-up, the POEM group had a better remission rate than the FCARMS group (92% vs 76%, 90% vs 61%, *p* = 0.03, 0.00 < 0.05) (Table 3). In 2 years follow up, barium esophagram grade I has the highest remission rate (*p* = 001, 0.00, 0.03 < 0.05) (Table 4). Overall analysis, there is no difference in curative effect between each type of Ling’s classification (*p* = 0.37, 0.67, 0.63 > 0.05) and between each type of HRM classification (*p* = 0.20, 0.58, 0.94 > 0.05). HRM classification, the remission rate of type II at 2 years in the POEM group (100%) is higher than that of the FCARMS group (64.3%) (*p* = 0.02), and the remission rate of HRM III in the two groups is equivalent (*p* > 0.05), not as good as HRM II (Table 5).

**Table 1:** The two groups of achalasia patients’ general information before treatment by propensity sore match

	Stent group (n = 100)	POEM group (n = 50)	*p*
Age (year)	40.09 ± 14.94	41.20 ± 12.86	0.65
Gender (male/female)	51/49	25/25	1.00
Course of disease (year)	5.81 ± 5.90	6.78 ± 7.83	0.44
Classification*			
HRM I/II/III (n)	4/28/29	0/14/19	0.31
Ling I/II/III (n)	58/40/2	30/20/0	0.60
Barium meal I/II/III(n)	54/35/11	20/23/7	0.27
Eckardt score	7.21 ± 1.78	6.96 ± 1.69	0.40
Dysphagia score	2.98 ± 0.14	2.98 ± 0.14	1.00

*Chi-square test, *T* test, *p* > 0.05, no significant difference

**Table 2:** The two group’s comparison of the operating time, fasting time, complications, hospitalized cost and symptoms response at 6 months after treatment

	Stent group (n = 100)	POEM group (n = 50)	*p*
Operating time** (min)	8.41 ± 4.88	73.94 ± 41.61	0.00
Fasting time (day)	2.24 ± 1.65	4.58 ± 2.50	0.00
Postoperative hospital stay (day)	5.73 ± 2.97	6.22 ± 2.50	0.32
Complications* perforated	0(0%)	6 (11.3%)	0.01
Bleeding	10 (10.0%)	2 (4.0%)	0.34
Fever	13 (13.0%)	10 (20.0%)	0.34
Chest pain	37 (37.0%)	14 (28.0%)	0.36
Abdominal pain	10 (10.0%)	10 (20.0%)	0.13
Nausea vomiting	30 (30.0%)	1 (2.0%)	0.00
Migration	25(25.0%)	–	–
Number of repetitions of gastroscopy	3.8 ± 2.4	2.1 ± 1.8	0.00
Acid reflux and heart burning	2 (2.0%)	0(0%)	0.00
The average hospitalization cost^#^ (¥)	17,787.85 ± 3711.69	27,705.41 ± 8868.09	0.00
Symptomatic response at 6 months			
Dysphagia score^##^	0.95 ± 0.92	0.58 ± 0.60	0.01
Weight-gaining (kg)	5.26 ± 3.04	5.75 ± 4.26	0.31

*T* test for Equality of means

*Fisher’s exact test, *p* > 0.05, no significant difference; *p* < 0.05, significant differences

^#^Expenses include the cost of nursing, evaluation, surgery, and medication during hospitalization. The cost of long-term follow-up should be taken into account. But follow-up is mainly through telephone or WeChat contact. This cost is so low that it is negligible compared to the cost of treatment, so it is not counted

**Operating times calculated: Operation time calculation for stent placement: the time between the first gastroscopic picture and the picture after the stent is placed; POEM operation time: the time between the esophagus incision and the complete clipping of the incision

^##^Dysphagia score is part of the Ecardt scores

**Table 3:** Curative efficacy comparison between two groups after treatment at 6-month, 1-year, and 2-year

Follow up data	FCARMS group (n = 100)		POEM group (n = 50)		*p*
	Remission	failure	Remission	failure	
6-month	89 (89.0%)	11 (10.0%)	47 (94.0%)	3 (6.0%)	0.39
1-year	76 (76.0%)	24 (24.0%)	46 (92.0%)	4 (8.0%)	0.03
2-year	61 (61.0%)	39 (39.0%)	45 (90.0%)	5 (10.0%)	0.00

Fisher’s exact test, *p* > 0.05, no significant difference; *p* < 0.05, significant differences

**Table 4:** Curative efficacy comparison among Ling’ subtype classifications, Barium meal grading and HRM classifications at 6-month, 1-year, and 2-year follow-up

	Ling’ classification (n)			*p*	Barium esophagram grade (n)			*p*	HRM classification (n)			*p*
	I	II	III		I	II	III		I	II	III	
6-month												
Remission	82	52	2	0.37	71	53	12	0.01	3	40	46	0.20
Failure	6	8	0		3	5	6		1	2	2	
1-year												
Remission	70	50	2	0.67	63	51	8	0.00	3	38	41	0.58
Failure	18	10	0		11	7	10		1	4	7	
2-year												
Remission	61	43	2	0.63	53	45	8	0.03	3	32	35	0.94
Failure	27	17	0		21	3	10		1	10	13	

Fisher’s exact test, *p* > 0.05, no significant difference; *p* < 0.05, significant differences

**Table 5:** curative effect comparison between FCARMS and POEM group in the same HRM classification at 6-month, 1-year, and 2-year follow-up

HRM classification	6-month		*p*	1-year		*p*	2-year		p
	Remission n (%)	Failure n (%)		Remission n (%)	Failure n (%)		Remission n (%)	Failure n (%)	
I									
FCARMS	3(75)	1(25)	–	3(75)	1(25)	–	3(75)	1(25)	–
POEM	0	0		0	0		0	0	
II									
FCARMS	26(92.9)	2(7.1)	0.55	24(85.7)	4(14.3)	0.28	18(64.3)	10(35.7)	0.02
POEM	14(100)	0		14(100)	0		14(100)	0(0)	
III									
FCARMS	29(100)	0(0)	0.15	25(86.2)	4(13.8)	1.00	19(65.5)	10(34.5)	0.20
POEM	17(89.5)	2(10.5)		16(84.2)	3(15.8)		16(84.2)	3(15.8)	

## Discussion

Achalasia is a primary esophageal motility disorder, characterized by aperistalsis in the distal portion of the esophageal body and incomplete or absent relaxation of the lower esophageal sphincter (LES). AS an incurable disease, the aim of treatment is to reduce LES pressure and solve the problem of dysphagia. Stent insertion treatment of achalasia was first described by De Palma in 1998 with encouraging results, originally used as a last resort in patients who had failed medical therapy or pneumatic dilation, or who were poor surgical candidates [26]. A study had reported that short-term stent placement in the treatment of achalasia is safe and effective, with good long-term clinical remission [15]. Undeniably, in this study, we found that the FCARMS implantation therapy has the advantages of short operation time, short fasting time, and low hospitalization costs. As a special expander, the stent has the characteristics of uniform force, which reduces the risk of perforation. Regarding the stent removal time, it has been reported in the previous literature that the average stent placement period can be sustained 3–6 weeks, or even 8 weeks if there are no obvious complications. Although the clinical effect is closely related to the stent expansion period, if the stent is inserted for more than 1 week, when the stent is retrieved, tissue proliferation around the stent can lead to more complications, such as pain or bleeding. In addition, the expansion of the stent for several consecutive days provides sufficient strength support for the esophageal wall, which has produced relatively good clinical effects. Therefore, we chose a stent insertion period of approximately 3–7 days. The stent upper line shrinks made it facilitate remove. Yet it can be reused when the disease recurs. The operation is simple and safe, and alleviate dysphagia quickly, besides, the hospital stay is short, so the cost reduced. These advantages are especially suitable for patients who are at high risk of anesthesia or who have a short survival period but urgently need to solve swallowing difficulties. Since the short-term effects, it requires more repetitions of gastroscopy. As the follow-up time is extended, the remission rate decreases, which may explain why the FCARMS group repeated endoscopy more often.

**Fig. 5:**
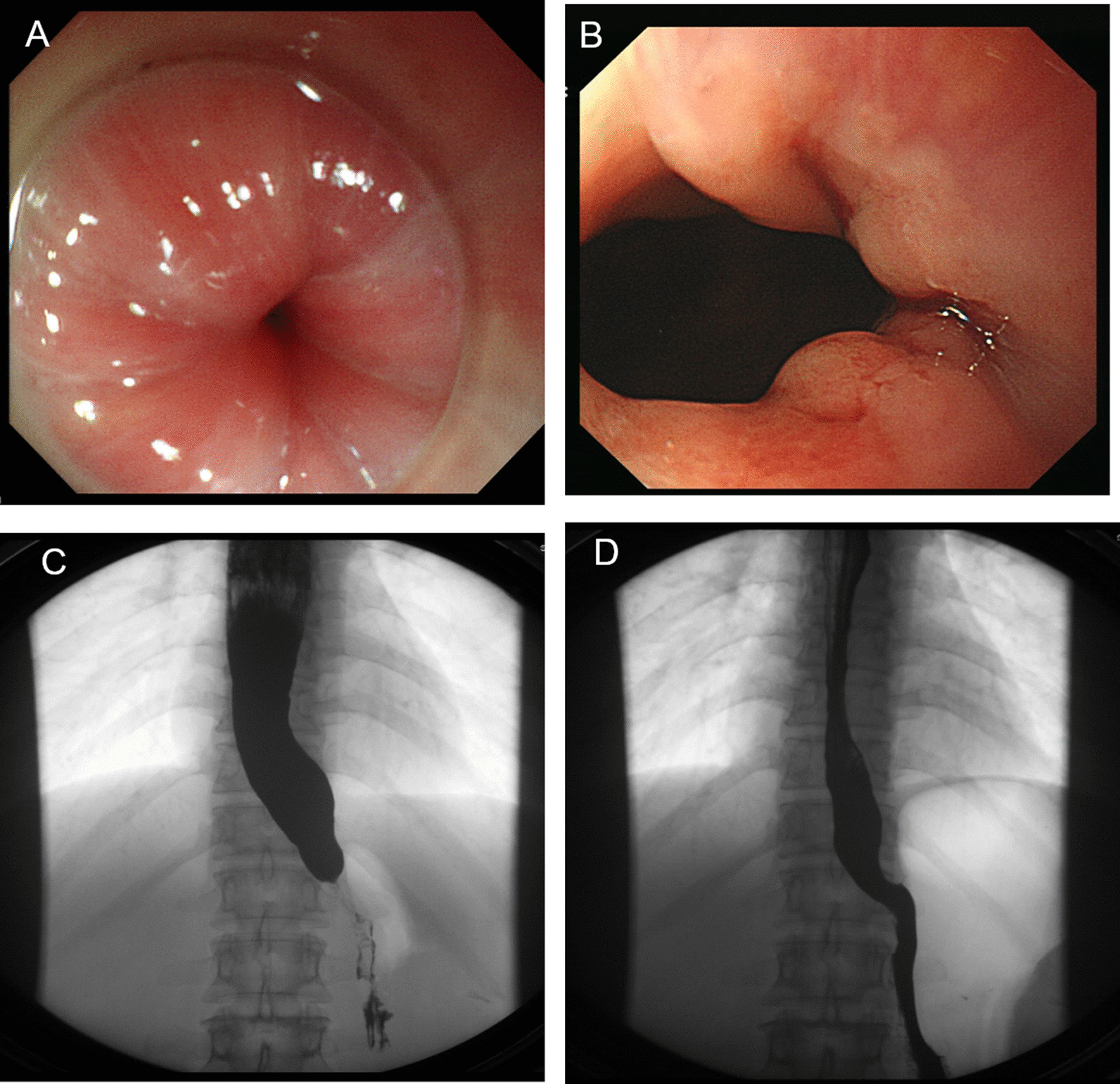
Typical pictures before and after receiving POEM treatment. **A** Cardia status before treatment under endoscopy. **B** Cardia status after treatment under endoscopy. **C** Barium retention in the lower esophagus before treatment. **D** Barium smoothly through the cardia after treatment

In this study, we adopt the PS matching analysis method to reduce the interference factors, making the research more accurate and the results more reliable. There is no significant difference in the number of the same HRM, Ling’s and barium esophagram subtype classification in the two groups. At 6 months, the dysphagia and weight-gaining were improved remarkable in both groups (Figs. 4, 5). At 1-year and 2-year, POEM had a much higher curative rate than FCARMS, the remission rate was 92.0% and 90.0%, respectively (*p* = 0.03, 0.00 < 0.05) (Table 3). For people with a long survival period more than 2 years, no risk of anesthesia, reluctance to repeat gastroscopy, or good economic conditions, POEM may be more appropriate, which still need further research.

It was reported that 30 mm diameter stents shift rate was 5.33%, much lower than our study (25%), if change the diameter may be a modified method [27]. In this study, patients received stent with diameter of 18–20 mm. The covered stent has a barbed needle to prevent the stent from moving up. So it falls into the stomach more often. Stent and POEM has equivalence effect at 6-month follow-up (*p* = 0.39 > 0.05) but as time goes, POEM had a better curative rate at 1-year and 2-year follow-up. Maybe it implies POEM has a better further long-term curative efficacy than stent. POEM has similar efficacy to first-line treatment of LHM, and the cure success rate is higher than that of PD [28]; the incidence of serious adverse events, but the incidence of GERD higher [29]. Recently, studies showed that the length of circular myotomy is related to the occurrence of GERD complications. Short myotomy is safer and more effective than standard myotomy, which can reduce GERD complications [30]. It is still need for further study to decrease complications and enhance curative effect. In our digestive endoscopy center, technology of POEM has carried out not long enough, but the stent implantation treatment has been developed for over 13 years. Through repeated short-time stent implantation, over 32% of patients’ have relieved dysphagia for more than 10 years and 100% over 5 years by repeated short-time stent implantation. They are still under further follow-up.

Similar to previous reported, Ling classification subtype I was the most common type of achalasia and account for 88% while subtype III was the least type and only 2% in this study. Yet it is a limitation to our study, in the LING’s type III, only two patients in the FCARMS group and zero in the POEM group. Similarly, in the HRM type I, only four patients in the FCARMS group and zero in the POEM group. A larger sample and a prospective study are required for more accurate and more meaningful data.

The remission rate of three grades of barium esophagram was also significant difference within 2 years follow-up (*p* = 0.01, 0.00, 0.03 < 0.05) (Table 4). Barium esophagram grade I had the best remission rate than other two grades, the curative efficiency reduced along with barium esophagram grading increasing. Barium esophagram grading according to esophageal maximal lumen diameter, which related to progress of the achalasia [25]. What imply earlier treatment curative effect is better. But the Ling’ subtype classifications didn’t affect remission rate (*p* = 0.37, 0.67, 0.63 > 0.05) (Table 4). Esophagogram Grade 3 with dilated esophagus, but the risk of esophagus was not increased due to the upward migration of the esophagus. The support of the stent only depends on the lower wall of the esophagus, which is sufficient to dilate the cardia, and the stent placement time is only 3 to 7 days. In this study, 11 cases of Esophagogram Grade 3, of which 2 cases had the stent moved up, the incidence rate was 18.2% (2/11), all were taken out under gastroscopy, and no serious adverse events were caused.

HRM classification type II achalasia used to be reported had the best response to PD and LHM treatment [31]. Data of this study is not different to previous report, in POEM group, we discovered that HRM II classification had the best remission rate among three subtypes within 2 years follow up (*p* = 0.20, 0.58, 0.94 > 0.05). In the FCARMS group, the highest clinical efficacy rate was 100% (29/29) of HRM type III and 92.9% (26/28) of HRM type II in stent group at 6 month follow-up (Table 5). It shows that within 1 year, HRM classification II, III, FCARMS and POEM treatment methods are all effective (Table 5). HRM classification II had the best response to POEM at 2 years follow up. HRM II in POEM group had a remission rate reached to 100% within 2 years follow-up. HRM type II is defined as achalasia with panesophageal pressurization. After POEM, the circular muscle of the lower esophageal sphincter was incised, the muscle relaxes, the pressure decreases, and the symptoms of dysphagia are relieved. This explains why HRM II is most suitable for POEM treatment.

As we known, HRM type III is featured with spasm, always considered most difficult to relieve [32], so the stent placed more proximally in HRM Type 3 than in other types. In this study we found FCARMS had a remission rate up to 100% in HRM type III. HRM Type 1 and Type 2 peristalsis disappears, and the closed state of the cardia was eliminated by stent treatment, and food enters the stomach through gravity, while HRM Type 3 has contraction, even if it is immature contraction, it is also a driving force, without cardiac resistance, so more effect. The above is just my guess, the answer to the question needs further research. In this study, HRM type III, the stent and POEM group had equivalence curative efficiency for 2 years follow-up. HRM III can be treated by both methods. As the stent needed only pay once, it can be sterilized and preserved after being removed, when dysphagia recurred can be reused, made more cost-effective. By contrast, stent implantation cost shorter operating time and less fee yet without severe complications. We believe that there are three kinds of people who are indicated for stent therapy. Firstly, for elderly patients, especially those with cerebrovascular disease cannot undergo long endoscopic operation and prolonged anesthesia but were badly in need of solution dysphagia, stent is an appropriate choice. Secondly, since stents are cheap and the incidence of GERD is low, for patients with financial difficulties who do not mind repeating procedures, FRARMS therapy is a good treatment option. Furthermore, for patients who want to resolve dysphagia, but have not decided to choose POEM or LHM surgery, stenting is an effective bridging treatment that does not interfere with the choice of other treatments.

## Conclusions

Stent placement is a cost-effective and safe treatment option for achalasia. The short-term effect (less than 6 months) of FCARMS is similar to that of POEM, the long-term effect (more than 2 years), POEM is better than FCARMS. HRM II is most suitable for POEM treatment. Our results indicate that Patients can choose treatment methods according to their own conditions.

## Data Availability

The datasets and materials used during the current study are available from the corresponding author on reasonable request.

## Abbreviations

FCARMS: Fully coated anti-reflux metal stent
POEM: Per-oral endoscopic myotomy
PS: Propensity score
LES: Lower esophageal sphincter
PD: Pneumatic dilation
LHM: Laparoscopic Heller myotomy
HRM: High-resolutionmanometry
GEJ: Gastroesophageal junction
